# Supplementary material for: MetaRibo-Seq measures translation in microbiomes
Source: Nat Commun. 2020 Jun 29;11:3268. doi: 10.1038/s41467-020-17081-z (PMC7324362; doi:10.1038/s41467-020-17081-z)
Supplement: Supplementary file 10 — Supplementary Data 7 [file 41467_2020_17081_MOESM10_ESM.zip › File2/Confidence_High_Taxonomy/133136_out.krona.html]

Javascript must be enabled to view this page.

members
magnitude
magnitudeUnassigned
count
unassigned
taxon
rank

133136\_out

16

superkingdom
16
2

phylum
16
976

class
16
200643

171549
order
16

815
family
1

816
genus
1

585543
species
1

SRS105082\_contig\_number\_22274

171552
family
15

838
genus
15


SRS012916\_contig\_number\_contig-100\_794.168664SRS012969\_contig\_number\_contig-100\_779.233981SRS012989\_contig\_number\_contig-100\_4161.58861.58861SRS015794\_contig\_number\_1579SRS018888\_contig\_number\_5140SRS043841\_contig\_number\_contig-100\_1556.73022SRS044535\_contig\_number\_contig-100\_1791.127633SRS045713\_contig\_number\_18487SRS049712\_contig\_number\_356SRS053356\_contig\_number\_contig-100\_1742.276708SRS064757\_contig\_number\_16299SRS104036\_contig\_number\_3348SRS143181\_contig\_number\_contig-100\_782.67880SRS143876\_contig\_number\_21082SRS144506\_contig\_number\_49614
165179
15
species
